# Increased diacylglycerol kinase ζ expression in human metastatic colon cancer cells augments Rho GTPase activity and contributes to enhanced invasion

**DOI:** 10.1186/1471-2407-14-208

**Published:** 2014-03-19

**Authors:** Kun Cai, Kirk Mulatz, Ryan Ard, Thanh Nguyen, Stephen H Gee

**Affiliations:** 1 Department of Cellular and Molecular Medicine, University of Ottawa, 451 Smyth Rd, Ottawa, ON K1H 8 M5, Canada; 2 Present address: Institute of Cell Biology, School of Biological Sciences, University of Edinburgh, 6.34 Swann Building, Edinburgh EH9 3JR, UK

**Keywords:** Colon carcinoma, Diacylglycerol kinase, Rho GTPase, Metastasis

## Abstract

**Background:**

Unraveling the signaling pathways responsible for the establishment of a metastatic phenotype in carcinoma cells is critically important for understanding the pathology of cancer. The acquisition of cell motility is a key property of metastatic tumor cells and is a prerequisite for invasion. Rho GTPases regulate actin cytoskeleton reorganization and the cellular responses required for cell motility and invasion. Diacylglycerol kinase ζ (DGKζ), an enzyme that phosphorylates diacylglycerol to yield phosphatidic acid, regulates the activity of the Rho GTPases Rac1 and RhoA. DGKζ mRNA is highly expressed in several different colon cancer cell lines, as well as in colon cancer tissue relative to normal colonic epithelium, and thus may contribute to the metastatic process.

**Methods:**

To investigate potential roles of DGKζ in cancer metastasis, a cellular, isogenic model of human colorectal cancer metastatic transition was used. DGKζ protein levels, Rac1 and RhoA activity, and PAK phosphorylation were measured in the non-metastatic SW480 adenocarcinoma cell line and its highly metastatic variant, the SW620 line. The effect of DGKζ silencing on Rho GTPase activity and invasion through Matrigel-coated Transwell inserts was studied in SW620 cells. Invasiveness was also measured in PC-3 prostate cancer and MDA-MB-231 breast cancer cells depleted of DGKζ.

**Results:**

DGKζ protein levels were elevated approximately 3-fold in SW620 cells compared to SW480 cells. There was a concomitant increase in active Rac1 in SW620 cells, as well as substantial increases in the expression and phosphorylation of the Rac1 effector PAK1. Similarly, RhoA activity and expression were increased in SW620 cells. Knockdown of DGKζ expression in SW620 cells by shRNA-mediated silencing significantly reduced Rac1 and RhoA activity and attenuated the invasiveness of SW620 cells *in vitro*. DGKζ silencing in highly metastatic MDA-MB-231 breast cancer cells and PC-3 prostate cancer cells also significantly attenuated their invasiveness.

**Conclusion:**

Elevated DGKζ expression contributes to increased Rho GTPase activation and the enhanced motility of metastatic cancer cells. These findings warrant further investigation of the clinical relevance of DGKζ upregulation in colon and other cancers. Interfering with DGKζ function could provide a means of inhibiting invasion and metastasis.

## Background

Colorectal carcinoma (CRC) is one of the leading causes of mortality in Western countries. The number of new cases in the United States was predicted to reach 103,170 in the year 2012, with 51,690 deaths expected [1]. CRC frequently metastasizes into a systemic disease, often invading the lymph nodes and other visceral organs. The occurrence of metastases due to tumour progression is responsible for the vast majority of cancer-related deaths.

The progression of normal colonic epithelium into adenoma and later, into malignant adenocarcinoma, is associated with diverse genetic and epigenetic alterations [2,3]. However, the vast majority of CRCs share a well characterized sequence of inactivating mutations in tumor suppressor genes and gain-of-function alterations in oncogenes [2,4]. The molecular pathways perturbed by these key genetic changes are relatively well understood. In contrast, there is limited information about the molecular changes that give rise to the subsequent stages of colorectal progression, from carcinoma *in situ*, to invasive carcinoma, to lymph node and visceral metastasis [5]. Understanding how CRC metastases develop is critical for the ultimate control of this cancer.

Metastasis is a complex process that begins with the invasion of cancer cells into the surrounding tissues. The acquisition of enhanced cell motility and invasiveness enable a tumor cell to break away from the primary site, enter and exit the circulation, and successfully establish a metastatic colony [6]. Cancer cells acquire migratory and invasive properties through disruption of cell-cell junctions, changes in focal adhesion complexes, and extensive reorganization of the actin cytoskeleton [7,8]. In mammalian cells, the generation of actin-based dynamic motile structures is regulated by the Rho family of small GTPases, of which the best studied members are Cdc42, Rac1 and RhoA. RhoA is involved in the maintenance of actin stress fibers and focal adhesions, Rac1 in the formation of lamellipodia and membrane ruffles and Cdc42 in the formation of filopodia [9,10]. The coordinated activation of these GTPases is thought to be required for efficient migration [11,12].

Mutations in Rho GTPases are rarely the cause of cancer; however the increased expression or activation of Rho GTPases is associated with a variety of cancer types [13], with enhanced invasion and metastatic potential, [14-18] and disease progression [19,20]. RhoA is overexpressed in colon carcinoma [19]. In contrast, Rac1 levels are normal in colorectal tumors, but truncated mutants of adenomatous polyposis coli (APC), which is the cause of sporadic and familial colorectal tumors [21], stimulate the activity of Asef, a Rac-specific guanine exchange factor [22]. The finding that Rho GTPase overexpression or hyperactivity, rather than activating mutations, are involved in human cancer suggests their regulatory proteins have a prominent role in deregulated signaling in cancer [23].

Rho GTPases cycle between inactive GDP-bound and active GTP-bound conformations, which enables them to function as binary switches. GTP-bound Rho proteins interact with multiple downstream effectors to elicit a variety of cellular responses including cytoskeletal reorganization, gene transcription, cell cycle regulation, and vesicular traffic [24]. The Rho GTPase cycle is tightly regulated by three classes of proteins. Guanine nucleotide exchange factors (GEFs) activate GTPases by promoting the exchange of GTP for GDP, whereas GTPase activating proteins (GAPs) inactivate Rho proteins by enhancing their intrinsic GTPase activity. Guanine nucleotide dissociation inhibitors (GDIs) prevent the dissociation of GDP and maintain the GTPases in an inactive state. GDIs also sequester Rho GTPases as soluble cytosolic complexes in which the C-terminal membrane-targeting lipid moiety of the GTPase is prevented from interacting with membranes [25,26]. Since the vast majority of Rho GTPase protein exists in a biologically inactive cytosolic complex with RhoGDI, this is a major point of regulation of Rho GTPase activity and function. A thorough understanding of the mechanisms that regulate Rho GTPases is therefore paramount for understanding deregulated Rho GTPase signaling in cancer.

Diacylglycerol kinases (DGKs) phosphorylate the lipid second messenger diacylglycerol (DAG) to yield PA. There are ten mammalian DGK isoforms (α, β, γ, etc.), each with specific patterns of expression, localization within cells and distinct structural domains [27]. Our previous studies demonstrated that DGKζ regulates both Rac1 and RhoA activation [28,29]. DGKζ forms two independent multiprotein signaling complexes with Rac1 and RhoA that function as selective RhoGDI dissociation factors. In DGKζ-deficient fibroblasts, Rac1 and RhoA activation are decreased and cell migration is significantly reduced [28,29]. In light of these findings, we hypothesized that increased DGKζ expression in cells would lead to enhanced Rho GTPase activity and increased migratory potential. DGKζ mRNA is highly expressed in colon cancer tissue relative to normal colonic epithelium [30]. To investigate potential roles of DGKζ in colon cancer metastasis, we used a cellular, isogenic model of human CRC metastatic transition. The SW480 and SW620 cell lines were established from biopsies taken at different intervals from the same 50-year-old CRC male patient [31]. SW480 cells derive from the primary tumor, a poorly differentiated (grade 4) CRC invading the muscularis propria. SW620 cells derive from a lymph node metastasis taken from the same individual six months later, when recurrent cancer with liver and mesenteric lymph node metastases was discovered.

Gene expression profile data available in the National Center for Biotechnology Information Gene Expression Omnibus (NCBI GEO) repository show the DGKζ transcript is increased in the metastatic SW620 cell line relative to the SW480 primary tumor line [5]. Here, we demonstrate that increased DGKζ protein levels in SW620 cells are associated with increased Rho GTPase activity and downstream signaling. Silencing of DGKζ expression in SW620 cells decreased Rac1 and RhoA activity and attenuated cell invasion. DGKζ silencing also attenuated the invasiveness of PC-3 prostate cancer and MDA-MB-231 breast cancer cells. Collectively, our findings suggest interfering with DGKζ function or expression may be a potential route to block the invasiveness of metastatic cancer cells.

## Methods

### Cell lines and culture conditions

Human colorectal tumor cell lines SW480 (ATCC CCL-227) and SW620 (ATCC CCL-228), prostate cancer cell line PC-3 (ATCC CRL-1435), and breast cancer cell line MDA-MB-231 (ATCC HTB-26) were obtained from the American Type Culture Collection (ATCC, Manassas, VA). The cells were verified according to ATCC Technical Bulletin No. 8 (2008) and grown at 37°C, 5% CO_2_ in Dulbecco’s modified Eagle’s medium (DMEM) supplemented with 10% fetal bovine serum (FBS), 2 mM L-glutamine, 100 units/ml penicillin and 100 μg/ml streptomycin.

### Antibodies

Affinity-purified antibody to the N-terminus of DGKζ was prepared from DGKζ antisera and has been thoroughly characterized [28,32-34]. Monoclonal Rac1 antibody (Catalogue number: 610650) was purchased from BD Biosciences (San Jose, CA). A polyclonal antibody to PAK1 (Catalogue number: 2602) was purchased from Cell Signaling Technologies (Danvers, MA). Anti-pPAK1 was a gift from Dr. Jonathan Chernoff (Fox Chase Cancer Center, Philadelphia, PA) [28,35]. Monoclonal anti α-tubulin antibody (Catalogue number: T5168) was purchased from Sigma-Aldrich (St. Louis, MO). Horseradish peroxidase-conjugated anti-rabbit (Catalogue number: 711-035-152) and anti-mouse (Catalogue number: 715-035-150) secondary antibodies were from Jackson ImmunoResearch Laboratories, Inc. (West Grove, PA).

### Establishment of DGKζ-knockdown SW620 and PC-3 cell lines

A lentiviral vector containing a small hairpin RNA (shRNA) construct targeted to human DGKζ gene (Catalog no. RHS3979-9569052) and a pLKO.1 empty lentiviral vector were purchased from Open Biosystems. The empty pLKO.1 vector (Catalog #RHS4080) contains a 18 bp stuffer sequence between the AgeI and EcoRI restriction sites. The shRNA oligonucleotides (oligo ID: TRCN0000000668, Open Biosystems) corresponding to the sequence on human DGKζ gene are: sense, 5′ TCG CAC AGG ATG AGA TTT ATA 3′; antisense, 5′ TAT AAA TCT CAT CCT GTG CGA 3′. Ultra-pure plasmid DNAs were prepared according to the manufacturer’s protocol. To generate stable knockdown cell lines, SW620 cells were transfected with the shRNA vector using FuGENE 6 Transfection Reagent (Roche-applied-science). After transfection, cells were incubated for 24 h. Transfectants were then selected with 7 μg/ml puromycin (Cellgro Catalog no. 61-385-RA). After two weeks, the stable clones were transferred to 96-well plates using sterile cloning discs (Bel-Art Products), grown until confluent, and then transferred to 60 mm cell plates. DGKζ levels in various clones were analyzed by immunoblotting. Clones with DGKζ protein levels that were substantially reduced compared to the controls were selected and maintained in medium containing 7 μg/ml puromycin. SW620 cells stably transfected with pLKO.1 empty vector were used as a control. DGKζ knockdown PC3 cell lines were generated in the same manner using Attractene transfection reagent to transfect the cells and 2.2ug/ml puromycin for selection.

### Lentiviral knockdown of DGKζ expression in MDA-MB-231 cells

A set of 3 lentiviral vectors containing shRNA targeted to the DGKζ gene (Thermoscientific; Catalogue no. RHS4531-EG8525) were used to generate lentivirus using the second generation packaging plasmids pMD2.G and psPAX2 from Addgene. MBA-MD-231 cells were infected with mixture of the 3 lentivirus and incubated for 30 hours at 37°C with 5% CO_2_ before being used in the invasion assays or extracted for western analysis.

### Rac1 activity assay

Levels of active Rac1 and RhoA were measured using a GST-PAK1 PBD and GST-Rhotekin RBD pull-down assay, respectively [36]. Cells were immediately harvested in chilled lysis buffer (50 mM Tris–HCl, pH 7.4, 150 mM NaCl, 1% Triton X-100, 20 mM MgCl_2_, and protease inhibitors). Lysates were centrifuged at 12,000 × *g* for 5 min. Equivalent amounts of protein were incubated with GST-PBD or -RBD beads for 30 min at 4°C. The beads were washed with lysis buffer, boiled in reducing sample buffer, and eluted proteins assayed for bound Rac1 or RhoA by immunoblotting.

### Western blot

Cells were lysed in an ice-cold lysis buffer (50 mM Tris–HCl, pH7.5, 150 mM NaCl, 50 mM MgCl_2_, 1% Triton X-100, 1 μg/ml antipain, 1 μg/ml pepstatin, 1 μg/ml leupeptin, 0.5 mM AEBSF, and 1 mM benzamidine hydrochloride). Cellular debris was removed by centrifugation (14,000 × g for 10 min at 4°C). Total protein concentration of the supernatants was determined using a colorimetric assay method (Bio-Rad). 100 μg of total protein from each sample was resolved by SDS-PAGE, transferred onto PVDF membrane (Millipore), immunoblotted with the affinity-purified polyclonal antibodies to DGKζ (1:100) and horseradish peroxidase-conjugated goat anti-rabbit secondary antibodies (1:800), and detected using enhanced chemiluminescence (Pierce Biotechnology). Differences in protein loading were monitored by probing membranes with monoclonal anti-α-Tubulin antibody.

### Invasion assay

Cellular invasive ability was evaluated using Corning 6.5 mm Transwell inserts (8 um pore size, 24 well plate, Fisher Scientific). For the SW620 and SW480 cell lines, the upper surface of the inserts was coated with 100 ul of 500 ug/ml Matrigel and the underside was treated with either 15 ug/ml (SW480 versus SW620) or 100 ug/ml (vector control versus shRNA) collagen type I. The cells were serum starved for 24 hours in DMEM containing 0.25% FBS, then re-suspended in DMEM/0.25% FBS/20 mM HEPES [pH 7.5] and seeded at 50,000 cells per insert. The medium in the lower chamber consisted of 600 ul DMEM/20% FBS/20 mM HEPES [pH 7.5], and 10 ug/ml collagen type I as chemoattractants. The cells were incubated at 37°C in a humidified atmosphere containing 5% CO_2_ for approximately 70 hours. The PC-3 cell lines were starved in serum-free DMEM, then resuspended in DMEM/0.1% FBS/20 mM HEPES [pH 7.5], and 25,000 cells each were seeded on inserts coated with 50 ul of 2 mg/ml Matrigel on the upper surface and 15 ug/ml collagen type I on the lower surface. The media in the lower chamber was the same as for the SW620 and SW480 lines. MDA-MB-231 cells were seeded at 25,000 cells per insert in serum-free DMEM/20 mM HEPES [pH 7.5] on inserts coated with 50 ul of 1 mg/ml Matrigel on the upper surface and 15ug/ml collagen type I on the lower surface. The lower chamber contained DMEM/10% FBS/10 ug/ml collagen type I. Both the PC3 and the MDA-MB-231assays were incubated for 24 hrs. Following incubation, the Matrigel was removed from the upper chambers using a cotton swab and the cells were fixed with 4% paraformaldehyde in PBS for 10 minutes, permeabilized with 0.5% Triton X-100 in PBS for 15 minutes, and stained with 30 ug/ml propidium iodide in PBS with 0.03% Triton X-100 for 6 hours. To compare the invasiveness of the cell lines, the inserts were placed on 24 × 50 mm coverslips and imaged on a Zeiss Observer D1 microscope fitted with a 10× objective. For each insert, five fields of view were imaged in a cross pattern and the number of invading cells counted. The counts were then averaged to obtain an invasive index. For each invasion assay plate, the invasive index of the inserts was normalized to the vector control cell lines (or to the SW620 cell line when compared to the SW480 line). Two control SW620/vector and two SW620/shRNA knock down lines were compared as well as three PC-3/vector and three PC-3/shRNA lines. Lentiviral shRNA infected verses vector infected MDA-MB-231 cells were also compared and the average reduction in DGKζ expression following infection was determined by western analysis.

## Results

### DGKζ expression is increased in a model of colorectal cancer progression to metastasis

**Figure 1:**
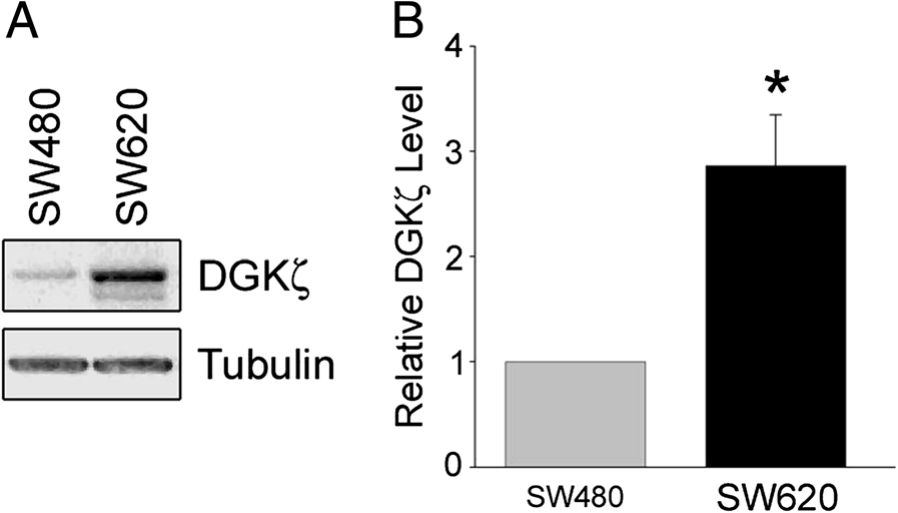
**DGKζ is increased in metastatic SW620 cells relative to non-metastatic SW480 cells. (A)** Detergent extracts prepared from lysates of SW480 and SW620 cells were analyzed by immunoblotting with an affinity-purified anti-DGKζ antibody (*top*) and an anti-α-tubulin monoclonal antibody (*bottom*). **(B)** Graph showing the quantification of DGKζ levels by densitometric analysis of immunoblots. The data were normalized to the level of α-tubulin and expressed as a fold increase relative to the amount in SW480 cells. Values are the average ± S.E.M. of four independent experiments. The asterisk indicates a highly significant difference (P < 0.01) from SW480 cells by Student’s *t*-test.

Analysis of endogenous DGKζ protein expression in SW480 and SW620 cells by immunoblotting cell lysates with an affinity purified anti-DGKζ antibody revealed a marked increase in DGKζ expression in SW620 cells (Figure 1A). Quantification of the band intensities after normalization to tubulin levels revealed DGKζ was elevated ~ 3-fold in SW620 cells (Figure 1B). These data are consistent with the increased DGKζ mRNA polysomal recruitment in SW620 cells compared to SW480 cells [5].

### Increased Rho GTPase activation in SW620 cells

**Figure 2:**
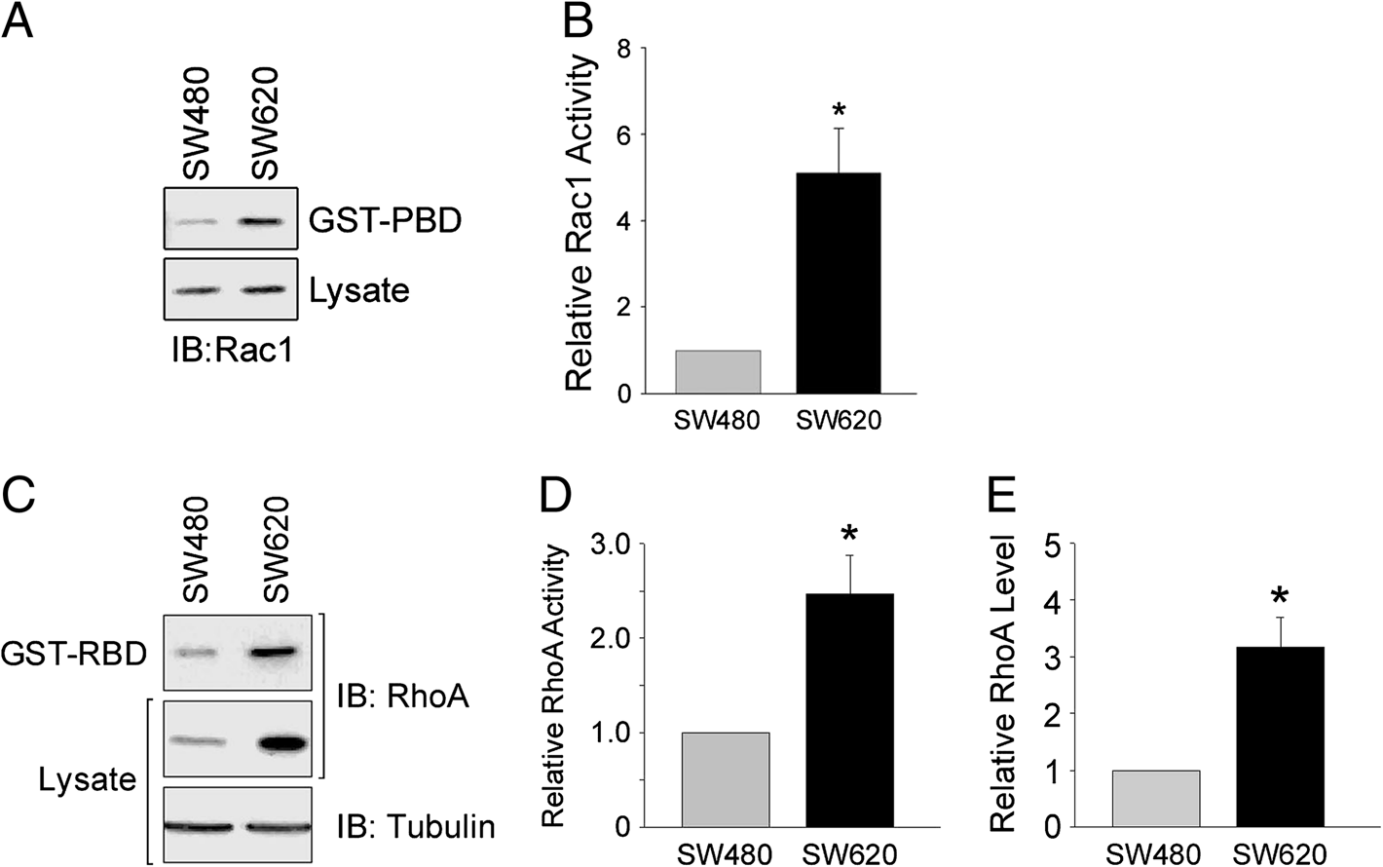
**Increased Rac1 and RhoA activity in SW620 cells relative to SW480 cells.** Detergent extracts from SW480 and SW620 cells were incubated with immobilized GST-PBD **(A)** or GST-RBD **(C)** and the bound proteins were analyzed by immunoblotting (IB) for Rac1 or RhoA, respectively (*top panels*). Total Rac1 and RhoA levels in the cell lysates are shown below. Tubulin is shown for comparison with RhoA. **(B and D)** Quantification of active Rac1 and RhoA levels, respectively, by densitometric analysis of immunoblots. The data were normalized to the amount of total Rac1 or tubulin (for RhoA) and are expressed as a fold increase relative to SW480 cells. **(E)** Graph showing the relative levels of RhoA in SW480 and SW620 cells. In each case, values are the average from at least three independent experiments ± S.E.M. The asterisks indicate a significant difference from SW480 cells (P < 0.05) by Student’s *t*-test.

Since DGKζ contributes to Rac1 and RhoA activation by promoting their release from RhoGDI [28,29], we next determined if the increased DGKζ expression in SW620 cells results in increased Rac1 and RhoA activity. The levels of GTP-bound Rac1 and RhoA were measured using effector pull-down assays, with the GTPase binding domains of PAK1 and Rhotekin, respectively [36]. There was a significant increase (approximately 5-fold) in Rac1 activity in SW620 cells despite the fact that total Rac1 levels were unchanged (Figure 2A and B). RhoA activity was increased approximately 3-fold, mirroring a similar increase in total RhoA protein levels (Figure 2C, D and E). Thus, the increases in active Rac1 and RhoA in SW620 cells parallel the increased DGKζ expression.

### Increased expression and phosphorylation of PAK1

**Figure 3:**
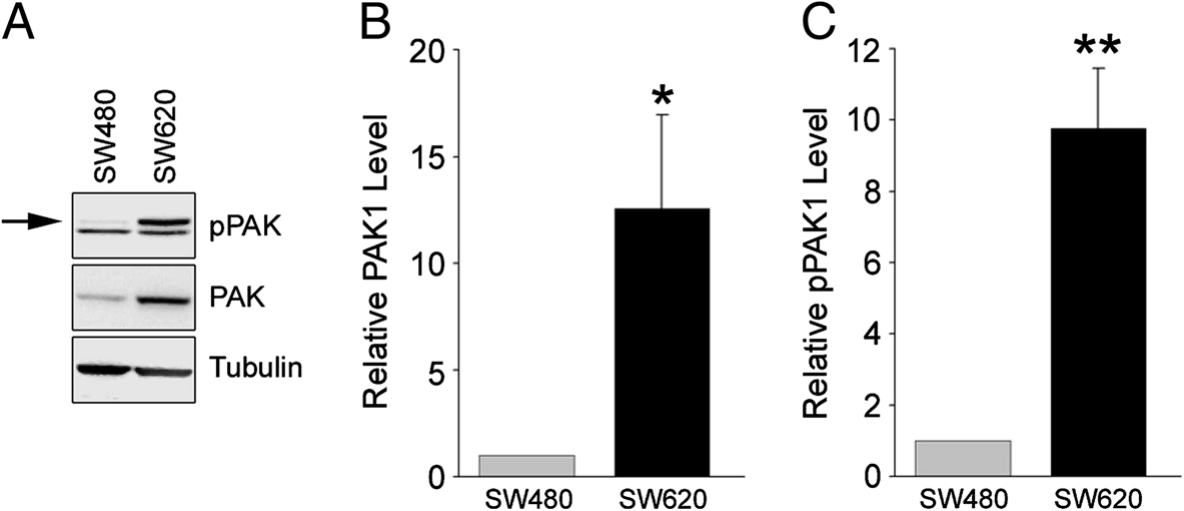
**Increased PAK1 expression and phosphorylation in SW620 cells. (A)** Detergent extracts prepared from SW480 and SW620 cells were analyzed by immunoblotting for phosphorylated (p)PAK1, total PAK1 and tubulin. **(B)** Graph showing the quantification of total PAK1 levels by densitometric analysis of immunoblots. **(C)** Quantification of pPAK1 levels. The intensity of the upper pPAK1 band (arrow in A) was measured and normalized to the level of tubulin. Values are the average ± S.E.M. of four independent experiments. One asterisk denotes a significant difference (P < 0.05) and two asterisks, a highly significant difference (P < 0.01) from SW480 cells by Student’s *t*-test.

The p21-activated kinases (PAKs) are Ser/Thr protein kinases whose activity is regulated by binding of active Rac or Cdc42 [37]. Since Rac1 activity was increased in SW620 cells, we evaluated whether PAK1 activity was similarly increased. Binding of active Rac1 or Cdc42 to PAK1 relieves autoinhibition and stimulates autophosphorylation, leading to increased kinase activity [37]. To evaluate PAK1 activity in SW480 and SW620 cells, detergent extracts of cell lysates were immunoblotted with a phospho-specific PAK1 antibody. Two pPAK1 bands were evident in SW480 cells; a faint upper band (Figure 3A, top panel, *arrow*), which represents hyper-phosphorylated PAK1, and a more prominent lower band, which is a less phosphorylated version [35]. The intensity of the hyper-phosphorylated band was significantly increased in SW620 cell lysates, while that of the lower band was unchanged, indicative of increased PAK1 phosphorylation in SW620 cells (Figure 3B). In contrast to Rac1, whose levels remained unchanged, total PAK1 levels were substantially (~10-fold) increased in SW620 cells (Figure 3A, bottom panel and Figure 3C). The increased PAK1 expression is consistent with the increase in polysomal PAK1 mRNA in SW620 versus SW480 cells [5].

### Establishment of SW620 cells with stable knockdown of DGKζ expression

**Figure 4:**
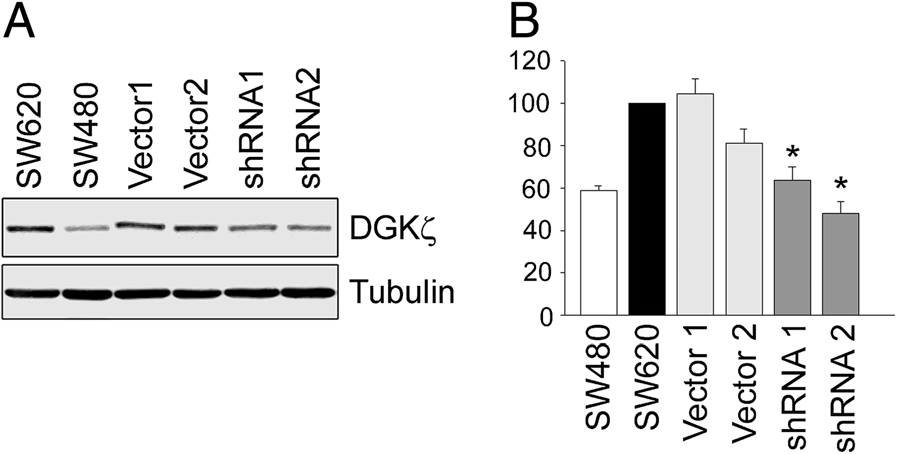
**Stable shRNA expression reduces DGKζ levels in SW620 cells. (A)** Equivalent amounts of protein from detergent extracts of SW620 cells, SW620 cells stably expressing an empty lentiviral vector (Vector1 and 2), or SW620 cells expressing an shRNA against DGKζ (shRNA1 and 2) were immunoblotted with anti-DGKζ (*top*) and anti-tubulin (*bottom*) antibodies. **(B)** Graph showing DGKζ protein levels in each cell line as measured by densitometric analysis of western blots. The data were normalized to the level of tubulin and are expressed as a percentage of DGKζ in SW620 cells. Values are the average of at least three independent experiments ± S.E.M. Statistical analysis was performed by a one-way ANOVA followed by a Tukey post-hoc multiple comparison test. The asterisks denote a highly significant difference (P < 0.001) from SW620 cells.

To investigate potential roles for DGKζ in the regulation of colon cancer cell invasion, we created stable SW620 cell lines that harbour either a lentiviral vector (pLKO) containing a short hairpin RNA (shRNA) sequence targeted to the DGKζ mRNA or the pLKO vector with no shRNA insert. Two control cell lines (SW620/Vector1 and 2) and two knockdown lines (SW620/shRNA1 and 2) were chosen for further analysis. Immunoblotting of cell lysates with the anti-DGKζ antibody revealed a marked decrease in DGKζ expression in the SW620/shRNA cell lines compared to the SW620/Vector lines Figure 4A. Quantification of immunoblots revealed average decreases of 40 and 50%, for the shRNA1 and 2 knockdown lines, respectively (Figure 4B), whereas the non-targeting shRNA vector control cells did not show significantly decreased DGKζ expression.

### Rac1 and RhoA activity are decreased by silencing DGKζ expression

**Figure 5:**
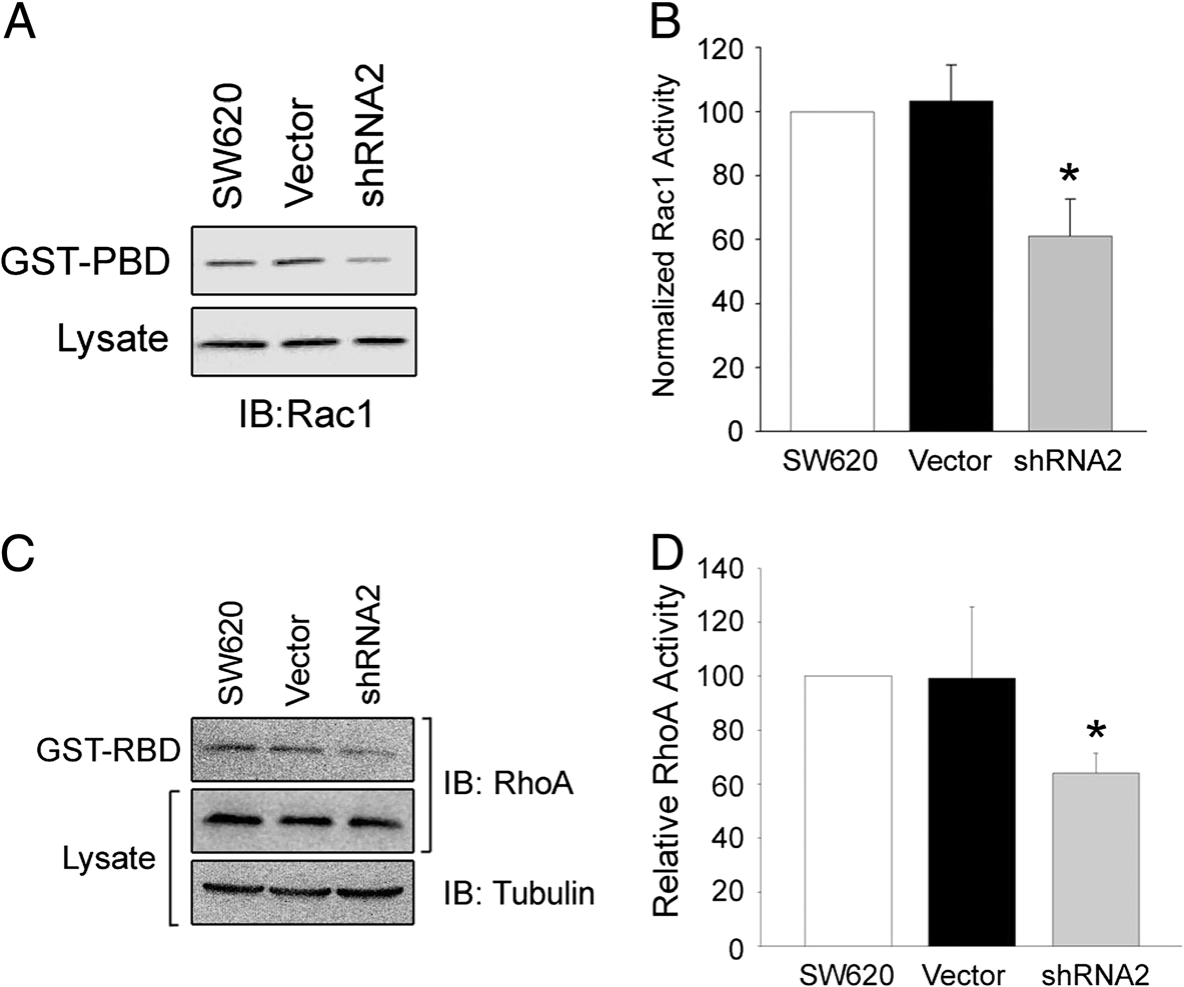
**Decreased Rac1 and RhoA activity in DGKζ knockdown cells.** Assay of global Rac1 and RhoA activity. Detergent extracts from the indicated cell lines were incubated with immobilized GST-PBD or GST-RBD and the bound proteins were analyzed by immunoblotting (IB) for Rac1 **(A)** or RhoA and tubulin **(C)**. Total Rac1 and RhoA levels in the cell lysates are shown. **(B and D)** Quantification of active Rac1 and RhoA levels, respectively, by densitometric analysis of immunoblots. The data were normalized to the amount of total Rac1 or RhoA protein and are expressed as a percentage of the activity in SW620 cells. Values are the average of at least three independent experiments ± S.E.M. The asterisks indicate a highly significant difference from SW620 cells (P < 0.005) by Student’s *t*-test.

Rac1 and RhoA activity were assayed in the stable cells lines. For these experiments, we chose the shRNA2 line because it showed the greatest reduction in DGKζ expression. The levels of GTP-bound Rac1 and RhoA were significantly decreased in SW620/shRNA2 cells, as compared with SW620 cells, while cells with non-targeting shRNA (SW620/Vector1) showed no significant change in either Rac1 or RhoA activity (Figure 5A-D). Notably, Rac1 and RhoA total protein levels were not affected by shRNA against DGKζ. These results suggest decreased DGKζ levels lead to decreased Rac1 and RhoA activity, but not expression.

### Decreased invasion of SW620 cells by stable knockdown of DGKζ

**Figure 6:**
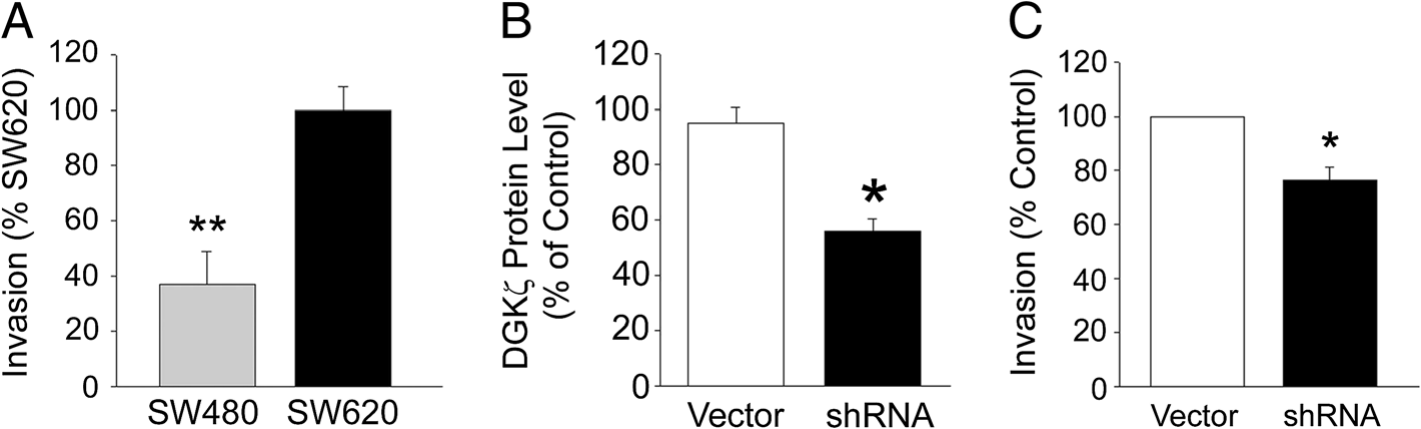
**Silencing DGKζ Expression Decreases Invasiveness of SW620 Cells.** SW480, SW620, SW620/Vector, and SW620/shRNA cells were placed in the upper chamber of a Transwell invasion plate and allowed to migrate across a Matrigel-coated, porous membrane for approximately 72 hrs. **(A)** Graph comparing the invasion of SW480 and SW620 cells. **(B)** Graph showing the average DGKζ level in SW620/Vector and SW620/shRNA cell lines normalized to the DGKζ level in SW620 cells. **(C)** Graph showing the average invasion of SW620/Vector and SW620/shRNA cell lines. Values are the mean ± S.E.M. from seven independent experiments. One asterisk indicates a significant difference (P < 0.05) and two asterisks, a highly significant difference (P < 0.005) from SW620 cells by Student’s *t*-test.

We recently reported that DGKζ-null mouse embryonic fibroblasts migrate less in two- and three-dimensional migration assays than their wild type counterparts [28]. To determine if silencing DGKζ expression affects the invasion of SW620 cells through a 3-D matrix, we compared SW480, SW620, SW620/vector and SW620/shRNA cells in an *in vitro* invasion assay. In agreement with previous reports [38-40], SW620 cells consistently invaded more through Matrigel-coated Transwell inserts than SW480 cells (Figure 6A). On average, the SW620/shRNA cells lines had DGKζ protein levels that were reduced by approximately 40% compared to SW620 cells, whereas SW620/Vector lines had DGKζ levels comparable to SW620 cells as expected (Figure 6B). The average invasion of the SW620/shRNA cell lines through the Transwell inserts was significantly less than SW620/vector cell lines (P < 0.05), suggesting siRNA-mediated silencing of DGKζ expression decreases the invasiveness of SW620 colon cancer cells (Figure 6C). Taken together, these results suggest DGKζ contributes to the overall invasive potential of SW620 colon cancer cells.

### DGKζ depletion decreases the invasiveness of prostate cancer and metastatic breast cancer cells

**Figure 7:**
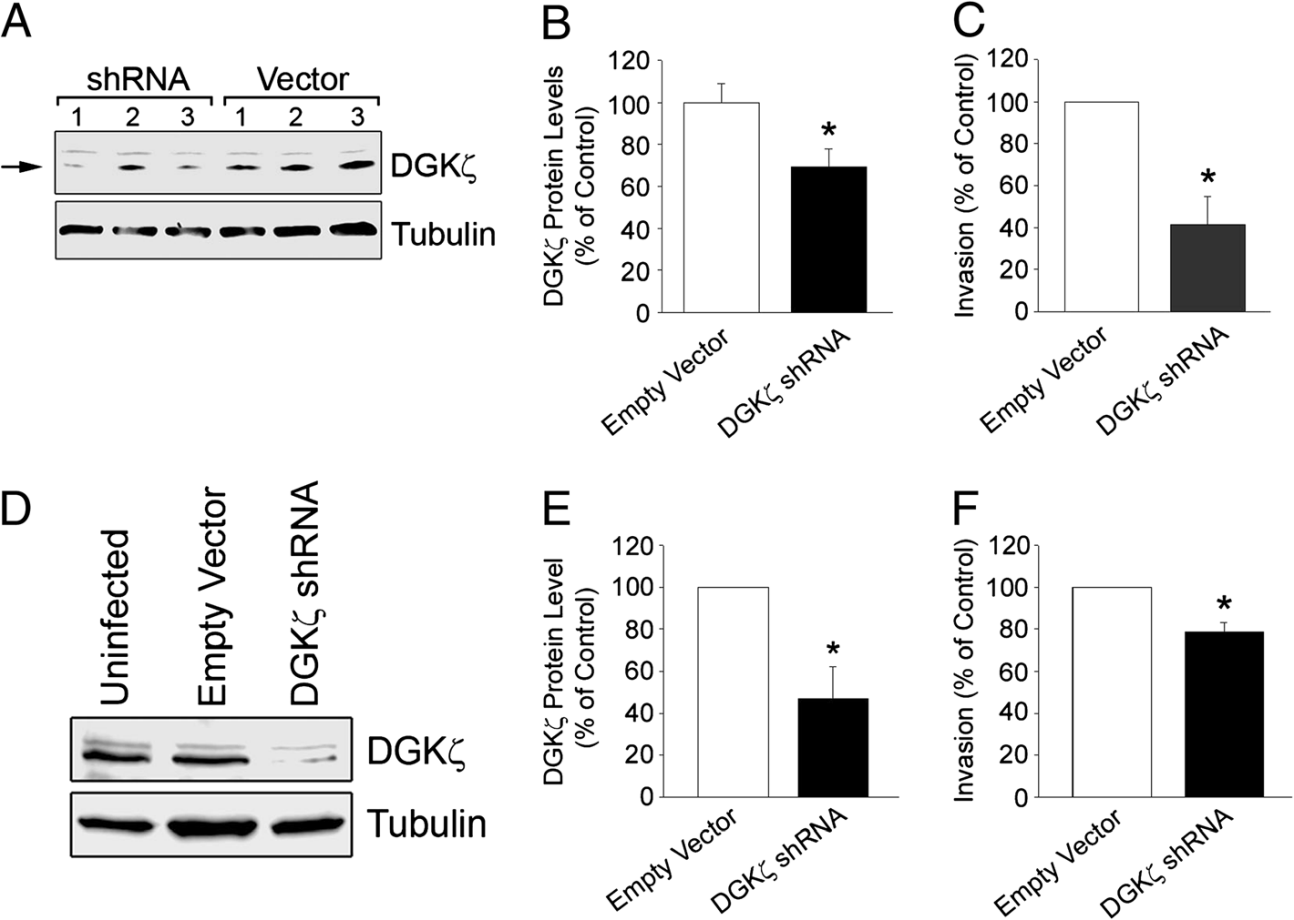
**DGKζ Silencing Attenuates Invasion of PC-3 Prostate and MDA-MB-231 Breast Cancer Cells. (A)** Western blot of DGKζ (*arrow*) in three PC-3/Vector and three PC-3/shRNA cell lines. Tubulin is shown for comparison. **(B and C)** Graphs showing the average DGKζ level **(B)** and invasion through Matrigel-coated Transwell inserts **(C)** of PC-3/Vector and PC-3/shRNA cell lines. The data were normalized to Empty Vector control cells. Values in **B** and **C** are the mean ± S.E.M. from five independent experiments. **(D)** Western blot of DGKζ in MDA-MB-231/Vector and MDA-MB-231/shRNA cells. Tubulin is shown for comparison. **(E and F)** Graphs showing the average DGKζ level **(E**) and invasiveness **(F)** of MDA-MB-231/Vector and MDA-MB-231/shRNA cells, normalized to the level in Empty Vector control cells. Values in **E** and **F** are the mean ± S.E.M. from four independent experiments. An asterisk indicates a significant difference (P < 0.05) from Vector cells by Student’s *t*-test.

To validate and extend the generality of our findings in colon cancer cells, DGKζ was depleted from two additional cell lines, the PC-3 prostate cancer line and the highly metastatic breast cancer line MDA-MB-231. Three stable cell lines each of PC-3/Vector and PC-3/shRNA were chosen for analysis. Western blotting of cell lysates revealed DGKζ expression was reduced in the PC-3/shRNA lines compared to PC-3/Vector lines (Figure 7A). Quantification of the western blot signals revealed an average decrease of approximately 40% in the shRNA lines (Figure 7B). The average invasiveness of the PC-3/shRNA was reduced by approximately 60% compared to PC-3/Vector lines (Figure 7C).

To silence DGKζ expression in MDA-MB-231 cells, three different DGKζ shRNA constructs cloned into the pGIPZ lentiviral vector were used to produce separate lentivirus stocks (see Materials and Methods). MDA-MB-231 cells infected with a mix of the three virus particles showed a substantial decrease in DGKζ expression (approximately 60%) compared to cells infected with the pGIPZ vector alone (Figure 7D and E). The invasion of MDA-MB-231/shRNA cells was significantly reduced compared to MDA-MB-231/Vector cells (Figure 7F). Collectively, the results from silencing studies in SW620, PC-3 and MDA-MB-231 cells strongly suggest DGKζ has a stimulatory role in cell invasion.

## Discussion

The development of metastatic tumors is a major cause of death in many human cancers. CRC progresses from adenoma to malignant adenocarcinoma to invasive carcinoma, and finally, to metastatic cancer. The origin of the SW480 and SW620 cell lines from the spontaneous progression of a human CRC in a single patient makes this a useful system for the analysis of gene expression changes during the transition from invasive carcinoma to metastasis. This isogenic, cellular model of CRC has been extensively validated and several studies showed SW620 cells are more invasive than SW480 cells *in vitro*[38,39]. In this study, we documented a ~3-fold increase in the level of DGKζ protein in SW620 cells, as compared to SW480 cells. Silencing of DGKζ expression by ~50% reduced the invasiveness of SW620 cells, suggesting DGKζ contributes significantly to the increased motility of this cancer cell line. Moreover, silencing DGKζ expression in PC-3 and MDA-MB-231 cells also lead to reductions in their invasiveness. Taken together, these findings strongly suggest DGKζ contributes to the overall invasive potential of SW620, PC-3 and MDA-MB-231 cells.

The significance of these findings relates to our previous work, which established DGKζ as a critical regulator of both Rac1 and RhoA activity [28,29]. In the former case, we showed DGKζ-derived PA activates PAK1, which phosphorylates RhoGDI, allowing for the release and subsequent Rac1 activation. Mouse embryonic fibroblasts deficient in DGKζ have reduced Rac1 activity and reduced Rac1-related structures such as lamellipodia and membrane ruffles [28]. Consistent with these findings, knockdown of DGKζ expression in SW620 cells significantly reduced Rac1 activity. Rac1 protein levels remained constant however, suggesting DGKζ acts primarily at the level of Rac1 activation. In DGKζ-null fibroblasts, even the complete absence of DGKζ only decreased Rac1 activity by ~ 50% suggesting other mechanisms contribute to Rac1 activation. Indeed, at least two additional DGK isoforms, DGKζ and DGKζ reportedly contribute to the regulation of Rac1 activation. DGKζ-dependent activation of atypical PKCζ/ζ mediates the release of Rac from RhoGDI in epithelial cells in response to hepatocyte growth factor [41,42], while DGKζ acts as an upstream suppressor of Rac1 activity in fibroblasts [43]. However, the polysomal mRNA expression of DGKζ or DGKζ was not substantially different in SW480 and SW620 cells and therefore the increased migration of SW620 cells is not likely due to changes in the expression of these isoforms.

RhoA activity was increased approximately 3-fold in SW620 cells compared to SW480 cells. Furthermore, there was a comparable increase in both RhoA and DGKζ expression. Since DGKζ is required for efficient RhoA activation [29], the combination of increased DGKζ and RhoA expression likely accounts for the increased RhoA activity in SW620 cells. However, since the level of RhoA activity but not protein was decreased by DGKζ silencing, it appears unlikely that DGKζ directly regulates RhoA expression. Thus, our findings in SW620 cells are consistent with our previous studies in mouse embryonic fibroblasts, which indicated that DGKζ regulates RhoA activity [29].

Activating mutations in Rho GTPases are rarely detected in human cancers. More frequently however, overexpression and/or hyperactivation of Rho proteins contribute to tumor progression and metastasis [19,20,44-48]. One study found that Rac1 plays a key role in the progression of CRC *in vivo*: decreased Rac1 expression blocked tumor formation in an orthotopic model of colorectal adenocarcinoma, whereas its overexpression in SW620 cells accelerated colorectal adenocarcinoma progression when the cells were injected into athymic nude mice [49]. In another study, RhoA activity correlated with lymph node metastasis in human colorectal cancer. More active RhoA in tumors with lymph node involvement than in those that did not metastasize suggests increased RhoA function is associated with enhanced tumor cell motility [46]. Together, these findings suggest decreasing Rac1 or RhoA expression, or alternatively, interfering with their ability to achieve or maintain the active GTP-bound state [50], is a viable strategy to reduce CRC progression and metastasis. The results presented herein suggest decreasing DGKζ expression or function is a potential route to reducing Rac1 and RhoA activity and the migratory ability of colon cancer cells. This strategy may be beneficial not only in cancers where DGKζ is overexpressed, but possibly also in cases where DGKζ is expressed at normal levels but Rac1 or RhoA are overexpressed or hyperactive.

Analysis of data deposited in the Oncomine database reveals DGKζ mRNA is highly expressed in several different colon cancer cell lines and in colon cancer tissue relative to normal colonic epithelium [30,51]. Moreover, DGKζ expression in CRC is high in comparison with other cancer types [52]. Thus, DGKζ and its downstream signaling pathways may be important factors influencing colon cancer progression. However, a limitation of our studies is the lack of correlative clinical data showing the DGKζ protein level is elevated in metastatic cancer. Thus, it will be important to validate our findings by comparing DGKζ protein levels in primary and metastatic tumor specimens. Moreover, the effect of silencing DGKζ expression on the *in vivo* metastatic potential of tumor cells with elevated DGKζ levels or high Rho GTPase activity remains to be investigated.

Finding that DGKζ is upregulated in other metastatic cancers would suggest interfering with its function might allow for a more general role in inhibiting tumor cell motility and invasion. DGKζ is also overexpressed several-fold in a variety of breast carcinoma and breast adenocarcinoma cell lines [53]. We found that silencing DGKζ expression in highly metastatic MDA-MB-231 cells decreased their invasiveness, suggesting DGKζ signaling also plays a role in the overall invasive potential of these cells. Similar results were obtained with PC-3 prostate cancer cells. Therefore, targeting DGKζ function in these cancers as well may provide new avenues for therapeutic strategies.

## Conclusion

In conclusion, our findings suggest DGKζ overexpression by colon carcinoma and other cancer cells plays an important role in tumor cell invasion, a key requirement for metastasis. Few molecular markers have proven to correlate well with stage and prognosis in colon cancer, particularly at later stages. It will be interesting to see if elevated DGKζ expression proves to be a predictive biomarker for patients with metastatic CRC or other cancer types.

## Abbreviations

ATCC: American Type Culture Collection; DAG: Diacylglycerol; DGK: Diacylglycerol kinase; DMEM: Dulbecco’s Modified Eagle Medium; FBS: Fetal bovine serum; PA: Phosphatidic acid; PAK1: p21-activated kinase 1.

## Competing interests

The authors declare that they have no competing interests.

## Authors’ contributions

KC, KM, RA and TN performed the experiments and analyzed the data. SG prepared the manuscript. All authors approved the final version of the manuscript.

## Pre-publication history

The pre-publication history for this paper can be accessed here:

http://www.biomedcentral.com/1471-2407/14/208/prepub
